# Revitalizing Bacillus Calmette–Guérin Immunotherapy for Bladder Cancer: Nanotechnology and Bioengineering Approaches

**DOI:** 10.3390/pharmaceutics16081067

**Published:** 2024-08-15

**Authors:** Maoxin Lv, Shihao Shang, Kepu Liu, Yuliang Wang, Peng Xu, Hao Song, Jie Zhang, Zelong Sun, Yuhao Yan, Zheng Zhu, Hao Wu, Hao Li

**Affiliations:** 1 Department of Urology, First Affiliated Hospital, Kunming Medical University, Kunming 650000, China; maoxin_lv@hotmail.com; 2 School of Basic Medical Sciences, Key Laboratory of Environment and Genes Related to Diseases of Ministry of Education, Xi’an Jiaotong University, Xi’an 710061, China; 3 Department of Urology, Xijing Hospital, Fourth Military Medical University, Xi’an 710032, China; shang225@hotmail.com (S.S.); sunzelong0413@hotmail.com (Z.S.);; 4 Department of Oncology, The Second Affiliated Hospital, Medical School of Xi’an Jiaotong University, Xi’an 710061, China; 5 Student Brigade of Basic Medicine School, Fourth Military Medical University, Xi’an 710032, China

**Keywords:** nano-based drug delivery systems, bladder cancer, immunotherapy, BCG, anticancer nanoparticles

## Abstract

Bacillus Calmette–Guérin (BCG) immunotherapy has been a cornerstone treatment for non-muscle-invasive bladder cancer for decades and still faces challenges, such as severe immune adverse reactions, which reduce its use as a first-line treatment. This review examines BCG therapy’s history, mechanisms, and current status, highlighting how nanotechnology and bioengineering are revitalizing its application. We discuss novel nanocarrier systems aimed at enhancing BCG’s efficacy while mitigating specific side effects. These approaches promise improved tumor targeting, better drug loading, and an enhanced stimulation of anti-tumor immune responses. Key strategies involve using materials such as liposomes, polymers, and magnetic particles to encapsulate BCG or functional BCG cell wall components. Additionally, co-delivering BCG with chemotherapeutics enhances drug targeting and tumor-killing effects while reducing drug toxicity, with some studies even achieving synergistic effects. While most studies remain experimental, this research direction offers hope for overcoming BCG’s limitations and advancing bladder cancer immunotherapy. Further elucidation of BCG’s mechanisms and rigorous safety evaluations of new delivery systems will be crucial for translating these innovations into clinical practice.

## 1. Introduction

Bladder cancer ranks as the fourth most prevalent cancer among men, representing 6% of new cancer diagnoses and 4% of cancer-related mortalities [1]. Bladder cancer is generally divided into two types based on the depth of invasion: non-muscle-invasive bladder cancer (NMIBC; Tis, Ta, and T1 stages) or muscle-invasive bladder cancer (MIBC; T2–T4 stages). NMIBC has a high recurrence rate, but its recurrence is often confined to the bladder [2]. MIBC recurrence may involve distant metastasis, making treatment more challenging and prognosis poorer [3,4]. The prognosis and treatment approaches differ significantly for the two types of bladder cancer. Current treatments for bladder cancer include surgery, chemotherapy, radiotherapy, targeted therapy, and immunotherapy [5].

At initial diagnosis, approximately 70% of bladder cancer patients are diagnosed with NMIBC which can be surgically removed via the transurethral resection of the bladder tumor (TURBT). However, 50–90% of NMIBC patients experience relapse within five years. Up to 21% of relapsed NMIBC will develop muscle invasion [5,6]. To reduce the risk of recurrence after TURBT, post-surgery bladder instillation therapy is a recommended strategy, significantly lowering the recurrence rate of bladder cancer [7]. Bacillus Calmette—Guerin (BCG) is the preferred medication for bladder instillation therapy. BCG, primarily an attenuated live vaccine used to treat tuberculosis, was first proposed in 1976 as an immunotherapy for bladder cancer [8]. It is used in bladder instillation therapy and has become the first-line treatment standard for NMIBC, increasing recurrence-free survival rates [9]. However, despite the initial success of BCG immunotherapy, 40% of patients eventually relapse after BCG instillation treatment [10,11]. Additionally, immune-related adverse events (irAEs) are common, with severe irAEs limiting benefits to only a portion of patients, leaving many to suffer from a lack of efficacy. Therefore, further research and improvements are needed to enhance treatment effectiveness.

The rapid development of nanotechnology and bioengineering has provided a new repurposing opportunity for this ancient drug. Utilizing redesigned BCG to create innovative drug delivery systems offers multiple solutions for the current challenges in bladder cancer immunotherapy. However, a comprehensive summary of these new delivery systems in bladder cancer immunotherapy is still lacking. This review aims to elucidate the current research status of BCG and the progress of various nanocarriers combined with BCG, highlighting their advantages and disadvantages focusing on their applications in bladder cancer immunotherapy.

## 2. The History and Current State of BCG Immunotherapy

BCG was developed in 1908 by Albert Calmette and Camille Guérin in France through the cultivation of *Mycobacterium bovis*. It is one of the earliest attenuated live vaccines used in humans. It remains the most widely used vaccine globally, with over 120 million doses administered annually, demonstrating its remarkable success in tuberculosis treatment [12]. In 1893, William Coley found that microorganisms could potentially treat cancer, sparking the exploration of using microbes in cancer treatment [13]. In 1924, the Pasteur Institute in Paris developed the first commercial BCG vaccines which began to be promoted globally for tuberculosis prevention [14]. Only four years later, Raymond Pearl’s research discovered a negative correlation between tuberculosis infection and cancer incidence, which further advanced the study of using Mycobacterium tuberculosis for cancer treatment [15]. In subsequent research, the repurposing of BCG for bladder cancer treatment achieved remarkable success.

In 1976, BCG was first used in immunotherapy for bladder cancer [8]. In 1980, the U.S. Food and Drug Administration (FDA) officially approved BCG for the treatment of NMIBC, marking a significant milestone for BCG immunotherapy in clinical practice. Thereafter, it rapidly became one of the standard treatments for superficial bladder cancer [16].

Numerous clinical trials have demonstrated that BCG effectively reduces the recurrence and progression of superficial bladder cancer, though notable adverse effects accompany it. The SWOG 8507 protocol (a 6-week induction followed by a 3-week maintenance at specified intervals over three years) shows better recurrence prevention than induction alone. Overall, BCG therapy achieves initial complete response rates of 55–65% for high-risk papillary tumors and 70–75% for Tis [10]. However, 25–45% of patients do not benefit initially, and 40% relapse despite initial success [11,17]. Though often minor, adverse effects of treatment, including common issues such as cystitis and flu-like symptoms, can cause significant intolerance in about 20% of patients. This intolerance may require adjustments in treatment schedules and, in some cases, treatment discontinuation. In the initial SWOG maintenance protocol, many patients withdrew, mainly due to irAEs, with only 16% of patients completing the planned maintenance therapy [10]. Currently, there is a lack of reliable biomarkers to predict which patients will respond well to BCG therapy, increasing treatment uncertainty [6]. For patients who do not respond to BCG therapy or have poor tolerance, more aggressive surgical treatments, such as radical cystectomy, are usually required. The application of BCG instillation therapy as a first-line treatment in bladder cancer is not satisfactory. Unfortunately, the immune mechanisms of bladder cancer have not yet been fully elucidated. Further clarification of these mechanisms is necessary to enhance understanding of this treatment approach and achieve more optimal therapeutic outcomes.

## 3. The Immune Mechanisms of BCG Bladder Cancer Treatment

**Figure 1:**
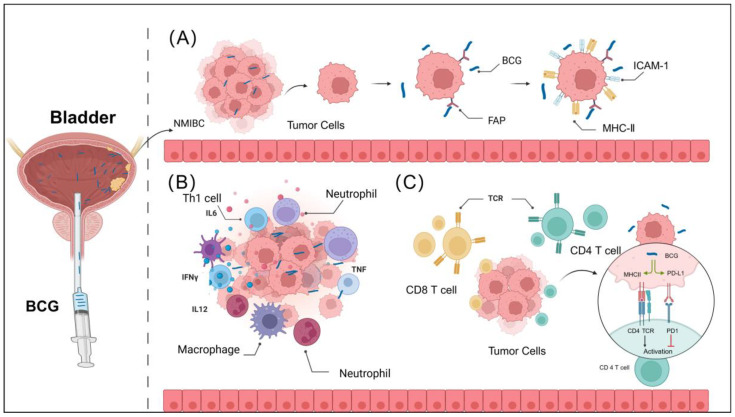
The immune mechanisms of BCG bladder cancer treatment. (**A**) Upon instillation, BCG adheres to the urothelium via fibronectin attachment protein (FAP), followed by internalization into tumor cells. This process leads to the upregulation of MHC-II and ICAM-1 expression in bladder cancer cells. (**B**) BCG promotes the infiltration of various immune cells into the bladder tumor tissue, releasing numerous inflammatory cytokines and chemokines, thereby altering the tumor immune microenvironment. (**C**) BCG activates adaptive immune cells, such as CD4 and CD8 T cells, which exert cytotoxic effects on bladder cancer cells. Additionally, BCG enhances PD-L1 expression on tumors, potentially improving the efficacy of immune checkpoint inhibitors. Abbreviations: BCG: Bacillus Calmette—Guerin. Created with Biorender.com (accessed on 28 June 2024).

Current research on the immune mechanisms of BCG suggests that the primary mechanisms by which BCG induces a robust immune response include several key aspects: the adhesion and internalization of BCG to the bladder epithelium, the promotion of innate immune activation, and the enhancement of adaptive immune activation (Figure 1). A deeper study and understanding of these mechanisms will greatly assist in the effective design of nano-immunodelivery systems.

### 3.1. BCG Attachment and Internalization in Bladder Urothelium

The bladder wall is composed of 5–7 layers of epithelial cells, with umbrella cells on the surface. Beneath these layers are fibrous connective tissue and blood vessels (lamina propria), thick muscle bundles (muscularis propria or detrusor muscle), and surrounding fat. The epithelial layer atop the lamina propria is called the urothelium, which covers the entire inner surface of the bladder. The urothelium serves as an impermeable barrier between the blood and urine, known as the bladder permeability barrier [18,19]. This strong barrier function makes the bladder well suited for urine storage but also limits the delivery of drugs for bladder cancer.

BCG binds to the urothelium through interactions between bacterial wall molecules and urothelial fibronectin. Bevers et al. described two mechanisms: (1) physicochemical interactions after damage to the glycosaminoglycan layer and (2) receptor–ligand-mediated attachment involving fibronectin. BCG’s antigen 85 and fibronectin attachment protein are crucial for this binding [20,21,22,23,24]. Fibronectin is present in the basement membrane and submucosa of the bladder wall, promoting BCG adhesion, especially in damaged areas such as cauterization sites [25]. However, other clinical evidence is inconsistent: warfarin users have higher risks of recurrence and progression, while aspirin users have lower risks. Other studies have found that fibrin clot inhibitors do not significantly impact BCG treatment outcomes, and thus the attachment mechanisms of BCG in humans remain unclear and difficult to verify [26,27]. BCG DNA expression can be detected in the bladder wall long after intravesical BCG instillation [23]. Additionally, persistent MHC class II expression has been observed in the urothelium following BCG therapy [28].

The uptake of BCG in the bladder remains unclear. BCG likely first encounters urothelial cells and is exposed to bladder-resident macrophages if there is tissue damage [29]. The internalization of BCG by urothelial cells is controversial. Bladder cancer cells, particularly those with highly genetic mutations, can internalize BCG via increased macropinocytosis, potentially involving receptor-mediated interactions with integrins and requiring fibronectin opsonization [30,31]. Normal urothelial cells generally do not internalize BCG, but high-grade tumor cells can; this process, dependent on the fibronectin opsonization of BCG, is inhibited by anti-β1 and anti-α5 integrin subunit antibodies [32]. Most studies are in vitro or in animals, highlighting the need for more human research.

### 3.2. BCG Enhances Innate Immune Activation

BCG immunotherapy induces both local and systemic immune responses [33,34]. Intravesical BCG, as a pathogen-associated molecular pattern (PAMP), activates various pattern recognition receptors (PRRs) on cell surfaces, including urothelial cells and antigen-presenting cells (APCs) [35], which produce cytokines and chemokines that attract granulocytes and mononuclear cells to the bladder. This activation leads to the production of cytokines and chemokines that attract granulocytes and mononuclear cells to the bladder. Current research has found that BCG primarily activates Toll-like receptors (TLRs) on the cell surface, which then activate the downstream MyD88 signaling pathway. This ultimately leads to the activation of nuclear factor kappa-B (NF-κB). Activated NF-κB translocates to the nucleus and promotes the transcription of cytokines involved in the immune response [36,37]. This leads to the formation of granulomas containing macrophages, dendritic cells, lymphocytes, neutrophils, and fibroblasts. Additionally, BCG stimulates cytokine production in urothelial carcinoma cell lines, including IL-6, IL-8, GM-CSF, and TNF. Human studies show increased urinary levels of cytokines like IL-1β, IL-8, IL-15, IL-18, CXCL9, CXCL10, GM-CSF, CCL2, and CCL3 after BCG instillation [38].

BCG immunotherapy can also induce both local and systemic immune responses. Intravesical BCG instillation activates urothelial cells and antigen-presenting cells (APCs), leading to cytokine and chemokine production that attract immune cells to the bladder. This results in the formation of granulomas containing various immune cells. BCG also upregulates cytokine production in urothelial carcinoma cell lines and induces cytokine and chemokine expression in human urine [39,40]. BCG can cause urothelial cells to express MHC class II, suggesting a role as APCs [41,42,43]. The resulting cytokine and chemokine expression recruits immune cells like neutrophils, monocytes, macrophages, T cells, B cells, and NK cells to the bladder [44,45,46,47,48,49].

Neutrophils play multiple roles in BCG immunotherapy, including acting as anti-tumor effector cells [50,51]. In a bladder cancer mouse model, neutrophil depletion reduced monocyte and CD4+ T cell infiltration, abrogated BCG’s therapeutic effect, and decreased survival. Neutrophils may act as anti-tumor effector cells through phagocytosis, generating reactive oxygen intermediates and releasing lytic enzymes and proapoptotic factors like TRAIL [52].

NK cells, part of the innate immune system, kill tumor cells in an antigen-independent manner. While modulating NK cell activity does not significantly affect mouse bladder cancer cell cytolysis or treatment efficacy, NK cell depletion reduces BCG immunotherapy efficacy and survival [53,54,55]. Thus, NK cells support BCG-induced cytotoxicity, though their role in human disease needs further exploration.

Repeated BCG instillations amplify the immune response through a prime-boost mechanism. While this induces a stronger anti-tumor effect, it can also lead to more intense side effects. Therefore, appropriately regulating the tumor microenvironment with BCG is considered a crucial therapeutic approach [56]. The study found that by blocking the COX2/PGE2/EP4 axis, it is possible to selectively enhance the recruitment of CTLs while reducing the attraction of Tregs and MDSCs. This selective modulation can improve the overall effectiveness of BCG therapy by promoting anti-tumor immunity and reducing immunosuppression within the TME [57]. The findings suggest new therapeutic strategies for enhancing BCG immunotherapy. Further research into the mechanisms is necessary to contribute to the design of more effective BCG delivery systems.

### 3.3. BCG Enhances Adaptive Immune Activation

BCG antigens are presented on APCs and urothelial cells via MHC class II molecules [41,42], interacting with CD4+ T cell receptors and leading to a TH1 immune response. This response, characterized by inducing IL-2, IL-12, IFNγ, TNF, and TNFβ [58], is associated with successful BCG immunotherapy. In contrast, a TH2 response, characterized by IL-4, IL-5, IL-6, and IL-10, correlates with BCG nonresponsiveness [59,60,61]. T cells are crucial for BCG’s anti-tumor effects [62,63]; the depletion of CD4+ or CD8+ T cells abrogates its efficacy. Increased CD4+ T cell counts and a higher CD4+ T cell ratio are linked to better patient outcomes. Repeated BCG instillations enhance CD4+ and CD8+ T cell infiltration into the bladder. BCG vaccination prior to BCG therapy may improve the therapeutic response, as indicated by better recurrence-free survival in patients with a positive PPD test. Therefore, some treatment strategies involve administering the BCG vaccine beforehand to enhance the effectiveness of BCG instillation therapy [64].

Programmed death ligand-1 (PD-L1) on tumor cells interacts with PD-1 on T cells, causing them to avoid attacking the tumor. Inhibitors targeting this checkpoint are important in tumor immunotherapy [65]. Wang et al. found that BCG upregulates PD-L1 on bladder cancer cells via the MAPK pathway and NF-κB activation. Combining anti-PD-L1 with BCG treatment increased tumor-infiltrating CD8+ T cells, decreased myeloid-derived suppressor cells, and improved tumor inhibition [66]. Max Kates et al. noted that PD-L1 contributes to BCG unresponsiveness due to pre-treatment adaptive immune responses and immune exhaustion [67]. High PD-L1 expression is linked to BCG failure, suggesting that combining BCG with anti-PD-L1 may be effective. Further verification is needed to confirm if BCG increases PD-L1 expression in tumor cells.

Systemic immune responses include increased cytokine and chemokine levels and lymphoproliferation [68]. Local and systemic adverse effects, such as cystitis and fever, often occur, sometimes requiring treatment with antituberculosis agents and corticosteroids [69]. Adverse effects can delay or stop treatment but generally do not correlate with efficacy [70]. Therefore, we need to adopt innovative approaches to enhance the benefits of BCG while minimizing its adverse effects.

## 4. Where Is BCG’S Future?

### 4.1. Challenges of BCG Use in Bladder Cancer Treatment

Aside from severe adverse reactions and patient tolerance issues hindering its development, BCG has also faced challenges due to production interruptions and market withdrawals. These issues have led to BCG supply shortages, resulting in treatment interruptions and forcing healthcare systems to seek alternative therapies, thereby lowering the clinical priority of BCG use and negatively impacting patient treatment standards [71,72]. Additionally, with the promotion of PD-L1/PD1 therapies, BCG has lost some of its prominence. Consequently, there is an urgent need to innovate BCG treatment methods to reduce adverse reactions and increase responsiveness in patients. However, the future of BCG may not be over yet; the development of nanotechnology presents the potential for its “repurposing” once again.

**Figure 2:**
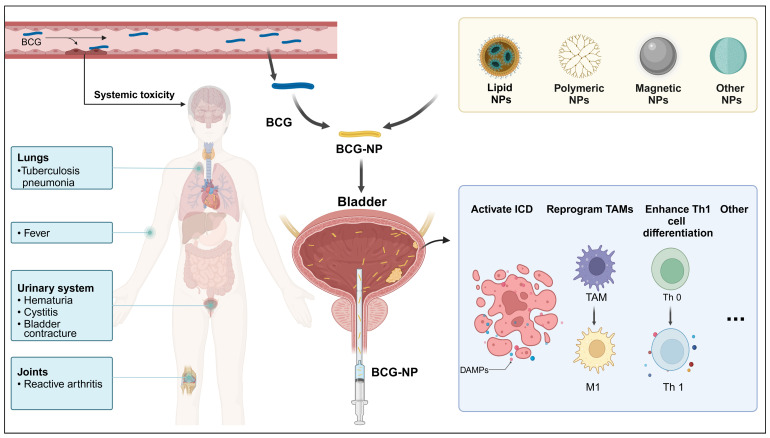
Adverse reactions and nanotechnology enhancements in BCG intravesical therapy for bladder cancer. BCG intravesical therapy for bladder cancer can cause adverse reactions such as hematuria, cystitis, fever, and reactive arthritis. Nanotechnology, including lipid NPs, polymeric NPs, and magnetic NPs, is being used to enhance BCG treatment. These modifications work by activating immunogenic cell death, reprogramming tumor-associated macrophages towards the M1 phenotype, and enhancing Th1 cell differentiation, thereby improving BCG efficacy and reducing adverse reactions. Abbreviations: BCG: Bacillus Calmette–Guerin, NPs: nanoparticles, ICD: immunogenic cell death, TAMs: tumor-associated macrophages. Created with Biorender.com (accessed on 28 June 2024).

Multiple attempts to improve BCG treatment are currently in progress. With the booming development of nanobiotechnology, optimizing BCG for novel nano-delivery systems in bladder cancer immunotherapy has reignited hope for BCG in treating bladder cancer. While the immune mechanism of BCG remains unclear, BCG immunotherapy has demonstrated the ability to induce a long-lasting and effective anti-tumor immune response, particularly yielding positive outcomes in bladder cancer treatment. As new targeted immunotherapies for various tumor types emerge and gain approval, reevaluating the cancer immune mechanisms triggered by BCG could inform the development of drugs that more effectively harness the immune system. Figure 2 illustrates the current predicament of BCG immunotherapy, the direction of its current development, and the potentially relevant immune mechanisms.

### 4.2. Nanotechnology and Engineered BCG

Since Richard Feynman first proposed the concept of nanotechnology in 1959 [73], nanotechnology has made rapid progress over the decades. Due to their various beneficial properties, nanomaterials have enormous potential as next-generation drug delivery carriers. Nanoparticles with different properties can deliver agents or drugs to specific cells within target organs via active or passive targeting [74]. These materials can be designed to extend the release of therapeutic drugs, thereby enhancing more sustained immune responses. Nanomaterials have been proven to effectively deliver immunotherapy drugs and induce immune responses in various immune cells. By recruiting the immune system to eliminate tumor cells, it is possible to activate previously hindered immune responses against cancer cells, potentially reducing the cytotoxic effects of traditional chemotherapy. There are numerous types of new nanomaterials, each with its own advantages and disadvantages. Here, our focus is primarily on the role of BCG in bladder cancer nano-immunotherapy delivery systems. Therefore, we will not provide detailed introductions to other materials. For further reading on this topic, we recommend consulting the relevant literature [75,76].

The advancement of nanotechnology has also driven the development of bacterial tumor therapy, especially advancing engineered bacterial delivery systems. With a deeper understanding of biology and microbiology, bacteria are considered promising delivery systems due to the discovery of differentiated evolutionary advantages in various bacteria suitable for anti-tumor therapy [77,78]. Specifically, many bacteria can penetrate tumor tissues that other drugs cannot reach through their self-propulsion capabilities [79,80,81]. Despite showing great potential in tumor therapy, natural bacteria clearly cannot meet the needs of tumor treatment [82]. The most obvious problem is that most bacteria are pathogenic and tend to cause severe cytokine storms and fatal side effects when entering human circulation [83]. Additionally, the anti-tumor capabilities of natural bacteria are insufficient to kill tumors, they are unable to meet therapeutic needs, and the treatment outcomes are not ideal. To enhance the anti-tumor therapeutic efficiency of naturally derived bacteria, it is necessary and effective to artificially engineer them, by specifically altering their functions to achieve improved anti-tumor effects [84].

### 4.3. Application of BCG in Novel Immune Delivery Systems

Novel nanotechnology has been employed to develop BCG-based drug delivery systems to achieve better therapeutic effects while reducing side effects. These systems have demonstrated improved tumor targeting, ultra-high drug loading, and effective anti-tumor immune stimulation [85,86]. Table 1 details the application of BCG-based nanoengineering technology in bladder cancer immunotherapy.

#### 4.3.1. BCG Bacterial Cell Walls: Advancing Drug Delivery

The history of bacterial therapy for cancer is extensive. To explore the use of bacteria in cancer treatment, early research delved into their immunological mechanisms. This included studies on the active components in BCG. By 1956, BCG was already considered an effective immunological adjuvant to enhance immune responses [96]. In 1963, it was discovered that the cell wall of mycobacteria acts as an adjuvant, primarily responsible for eliciting an immune response, a function not found in the centrifuged bacterial cytoplasmic contents [97]. Later, in 1971, Azuma and colleagues recognized the cell wall of BCG as a crucial component of the adjuvant, studying its adjuvant activity on immune responses [98]. Experiments demonstrated that specifically treated cell walls significantly enhanced both humoral and cellular immune responses against various antigens. The team further isolated the structure of the BCG cell wall by treating it with proteases and extracting it with organic solvents, thus separating a cell wall skeleton named “CWS-I” and a soluble part called “free lipids”. CWS-I, containing aggregated mycolic acids, arabinogalactan, and mucopeptide complexes, proved as effective in inhibiting tumor growth as the original cell wall, but could not promote regression in established tumors. However, when CWS-I was recombined with a component from the free lipids named P3, which includes mycolic acids and trehalose, it restored the full tumor-regressive activity of the original cell wall [99,100]. These findings advanced our understanding of the mycobacterial cell wall’s role in cancer and immune therapy, especially highlighting the specific bioactive components used in immunotherapy. Further animal studies validated these findings, establishing the great anti-tumor potential of the BCG cell wall and its effectiveness against various tumors in subsequent research [101,102,103,104,105]. Further studies also showed that different formulations of BCG cell wall (BCG-CW) affect its anti-tumor capabilities, sparking interest in designing various formulations of BCG-CW to enhance immunotherapy [106]. These studies have laid a solid foundation for the application of BCG in bladder cancer.

#### 4.3.2. Lipid Nanoparticles for Delivering Bacterial Cell Walls

In the early stages, a cell wall (CW) preparation composed of heat-killed BCG (BCG-CW) was developed using bioengineering methods, encapsulating BCG-CW into lipid particles. This cell wall preparation was incorporated into octaarginine-modified cationic liposomes (R8-liposomes-BCG-CW) [87]. Due to the instability and challenging preparation methods of BCG-CW, researchers further investigated the role of Bacillus Calmette–Guerin cell wall skeleton (BCG-CWS) in R8-liposomes-BCG-CWs for bladder cancer. They resolved the issue of delivering BCG-CWS effectively for bladder cancer therapy by developing a nanoparticulation method using lipid vehicles. This approach addressed the challenges associated with poor solubility, which hindered its clinical application. Encapsulating BCG-CWS in nano-sized lipid particles enhanced its dispersibility and uniformity, significantly improving its delivery and therapeutic efficacy against bladder cancer in cellular and animal models. This research provided a basis for the subsequent delivery of BCG-CWS and its effective fragments for bladder cancer immunotherapy [89]. Subsequent findings demonstrated that R8-liposomes-BCG-CWS could effectively inhibit bladder cancer. This structure increased the expression of NKG2D ligands on cancer cells, making them more susceptible to destruction by lymphokine-activated killer (LAK) cells. Results showed a significant upregulation of these ligands and an increased sensitivity of cancer cells to LAK cells, suggesting that R8-liposome-BCG-CWS could potentially improve the effectiveness of BCG immunotherapy for bladder cancer while reducing its side effects [88]. Furthermore, based on previous research, Tomoyuki Kato and colleagues found that heat-killed BCG exhibited antiproliferative activity against bladder cancer cells. Integrin α5β1 was identified as a potential biomarker for BCG’s direct effect on bladder cancer. Additionally, studies indicated that BCG-CWS inhibited bladder cancer cell proliferation [90]. The study by Nakamura et al. further investigated the mechanism behind the anti-tumor effect of BCG-CWS in bladder cancer. They found that the internalization of BCG-CWS by bladder cancer cells, with dendritic cells (DCs) not playing a major role in this process, is crucial for initiating an effective anti-tumor response. This challenges previous beliefs about the role of DCs and highlights the importance of direct interaction between BCG-CWS and cancer cells [107,108]. This suggested a potential method to enhance the safety of bladder cancer immunotherapy. Later research found that BCG binding to bladder cancer cells facilitated the internalization of delivered drugs by bladder cancer cells, thus proposing the delivery of functional penetrating peptides to reduce BCG toxicity. Research has shown that using the BCG surface peptide RWFV enhances the internalization of drugs in bladder cancer cells through a targeted, pH-sensitive lipid delivery system. This system efficiently transports immunotherapeutic oligonucleotides with minimal cytotoxicity. The RWFV peptide acts as a targeting ligand on the nanolipids, facilitating enhanced cellular binding, internalization, and trafficking within endosomal compartments. Composed of Cholesterol hemisuccinate (CHEMS) and 1,2-Dioleoyl-sn-glycero-3-phosphoethanolamine (DOPE), the nanoparticles release encapsulated CpG oligonucleotides in acidic endosomal environments, where they interact with TLR9 receptors to initiate a robust immune response. The absence of either the targeting ligand or the pH-sensitive properties results in less effective therapeutic outcomes, emphasizing the importance of incorporating both elements for successful delivery. Using key fragments of BCG instead of the full agent can retain targeting specificity while reducing immunological side effects, offering a valuable approach. Further, in vivo testing is required to evaluate this system’s potential as a BCG alternative for bladder cancer immunotherapy [91].

#### 4.3.3. Polymeric Nanoparticles

Building on the aforementioned research, more complex BCG delivery systems have recently been increasingly used for bladder cancer immunotherapy. The study by Erdoğar et al. explored using BCG loaded into cationic chitosan nanoparticles for bladder cancer treatment in rats. These nanoparticles aimed to improve BCG’s therapeutic efficacy by enhancing retention and absorption in the bladder. The findings showed that BCG-loaded nanoparticles significantly increased survival rates and reduced tumor growth compared to traditional BCG treatments. However, the study did not further investigate the underlying immune mechanisms [92]. To decrease recurrence rates following the transurethral resection of bladder tumors, Ma et al. synthesized a glutathione (GSH)-responsive lipophilic oxaliplatin prodrug, octadecyl-OXA-carboxylic acid, and incorporated it into cationic liposomes (LRO) containing a stearyl cell-penetrating peptide, C18-R8H3. The LRO formulation facilitated deeper penetration into bladder tumor tissues and released oxaliplatin via the reductant GSH in tumor cells. This method co-delivers oxaliplatin prodrug liposomes and low-dose BCG in a viscous chitosan solution (LRO-BCG/CS), enhancing the retention and penetration of chemotherapeutic agents in the bladder wall. Oxaliplatin induces immunogenic cell death (ICD), while BCG stimulates systemic anti-tumor immune responses. Even at low doses, this combination effectively triggers ICD, mitigates the immunosuppressive tumor microenvironment, and activates tumor-specific immune responses, significantly prolonging the survival of tumor-bearing mice. The minimal side effects indicate a promising and well-tolerated treatment strategy for bladder cancer patients. The dual targeting of tumors and the immune microenvironment has achieved significant therapeutic effects and presents potential for clinical application [93].

#### 4.3.4. Hybrid Formulations

**Figure 3:**
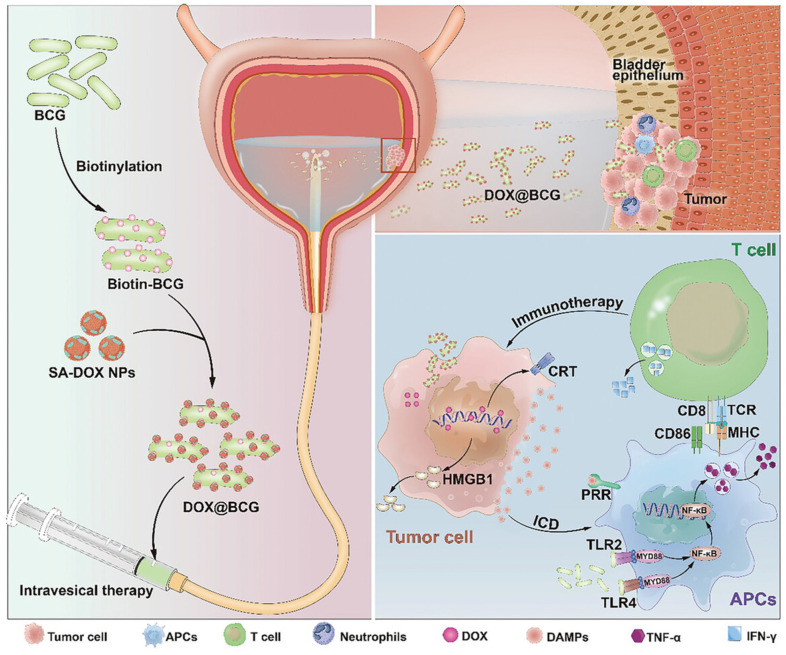
Schematic of using live BCG as a drug carrier for bladder cancer immunotherapy. Reprinted with permission from Liu et al. [95] from Wiley.

Magnetic nanoparticles have also been utilized in BCG-related delivery immunotherapy. A magnetic thermosensitive hydrogel was developed as an intravesical BCG delivery system, composed of chitosan, β-glycerophosphate (GP), and Fe_3_O_4_ magnetic nanoparticles (Fe_3_O_4_-MNPs). The magnetic injectable hydrogel significantly prolonged the retention time of BCG in the bladder under an external magnetic field. Compared to traditional BCG therapy for superficial bladder tumors, the hydrogel system-delivered BCG induced a stronger Th1 immune response and exhibited higher anti-tumor efficacy [94]. Transforming live BCG with nanomedicine linkage to form a new immunotherapy delivery system is a bold and innovative approach, showcasing the advanced capabilities of modern engineering technology. Liu et al. [95] developed an innovative co-delivery system that combines BCG with chemotherapeutic drugs. Employing the ‘biotin-streptavidin strategy’ to ensure stable linkage, they encapsulated DOX within live BCG bacteria (DOX@BCG) to enhance therapeutic efficacy. This system utilizes the natural adhesion of BCG to the bladder epithelium to precisely target DOX@BCG to tumor cells, thereby improving intratumoral drug transport. The synergy between BCG immunotherapy and DOX chemotherapy, as well as the ICD induced by DOX, has been proven effective. Simultaneously, BCG, as a pathogen-associated molecular pattern (PAMP), together with DAMP molecules, activates antigen-presenting cells (APCs), and presents antigens to CD8+ effector T cells, and induces intratumoral infiltration of T cells, M1 macrophages, and neutrophils. Finally, DOX-induced ICD of tumor cells and BCG together effectively establish anti-tumor immunity, thus playing a synergistic therapeutic role. Furthermore, this approach has shown improved tolerance and biosafety, and has established anti-tumor immunity within the tumor microenvironment, suggesting significant clinical translational potential for intravesical therapy in bladder cancer. Figure 3 illustrates a schematic of using live BCG as a drug carrier for bladder cancer immunotherapy.

## 5. Conclusions and Future Outlook

Nanotechnology and bioengineering aim to improve treatment responsiveness, prolong drug release, reduce side effects, and enhance immunotherapy effects by targeting tumors. Recently, many tumor-targeting nanodrug delivery systems have been developed to reduce toxicity [109], a design approach that has become widely achievable [110]. Due to the higher targeting efficiency of these nanodelivery systems, drugs can better accumulate in tumors, offering new perspectives for immunotherapy [111]. In clinical applications, various approved nanodrugs, such as albumin-bound paclitaxel [112], liposomal daunorubicin [113], and liposomal doxorubicin [114], exhibit fewer side effects compared to their original formulations. Consequently, higher doses of chemotherapy can be administered, providing significant insights for BCG immunotherapy in bladder cancer.

Although BCG immunotherapy for bladder cancer currently faces challenges and shows some decline in frontline use, new drug delivery methods such as nano delivery and engineered bacteria offer new hope for BCG’s continued application in immunotherapy. Benefiting from nanotechnology and engineered bacterial modifications, BCG has been further applied. Designing novel delivery systems that meet clinical needs by leveraging the characteristics of various nanomaterials and biomaterials has further advanced the development of bladder cancer immunotherapy. Research based on carriers of different nanomaterial properties or engineered bacterial designs reduces the toxicity of BCG through two approaches: (1) using functional bacterial wall components to enhance responsiveness while mitigating the irAEs caused by strong immune responses; and (2) using nanoengineering technology to co-deliver BCG with drugs, which shows synergistic effects and reduces the drug dosage. The above approaches can also be combined. These studies provide insights into our future development direction. However, the specific mechanisms of BCG have not been thoroughly elucidated, and it cannot exert its maximum potential in immunotherapy. The synergistic delivery schemes related to BCG are also relatively superficial and require more combinational trials. Recent studies have significantly advanced our understanding by identifying PD-L1 as a receptor that binds with fungi [115]. These findings strongly support the potential strategy of co-administering BCG with PD-L1 inhibitors.

Unfortunately, in the field of BCG nanodelivery, most related studies are still in the experimental stage. Applying these advanced drug delivery systems to clinical settings is a common challenge in nanodelivery tumor treatment, primarily due to the lack of reliable evidence regarding the safety of nanoparticles. The safety of some materials requires further investigation. Additionally, as research progresses, the structures of nanoparticles are becoming increasingly complex to achieve more functions, which hinders large-scale production. Furthermore, multifunctional nanoparticles entail higher manufacturing time and costs, which is a significant reason why few nanoparticles have entered clinical practice. We hope that more groundbreaking delivery systems will improve the treatment outlook for human bladder cancer in the future.

## Figures and Tables

**Table 1:** BCG-based nanoengineering in bladder cancer immunotherapy.

Base-Nanocarriers	Material	Therapeutic Agent	Advantages in Immunotherapy	Ref
Lipid nanoparticles	Liposomes	BCG-CW	Enhanced the internalization of BCG-CW into bladder cancer cells and induced an anti-tumor immune response	[87]
	Liposomes	BCG-CWS	Enhanced the expression of NKG2D ligands and targeting integrin α5β1 promoted the tumor uptake of CWS, activated APCs, and enhanced Th1 cell differentiation	[88,89,90]
	DOTAP+ DOPE+CHEMS	RWFV, CpG	Initiated a potent immune-stimulatory response and target macrophage, and activated APCs	[91]
Polymeric nanoparticles	Chitosan	BCG	-	[92]
	Chitosan	Oxaliplatin, BCG	Activated ICD, activated APCs, enhanced both cell-mediated and humoral immune response, and reprogrammed TAMs towards the M1	[93]
Magnetic nanoparticles	Fe_3_O_4_-MNP+ chitosan+ β-glycerophosphate	BCG	Enhanced the retention of BCG in the bladder and induced Th1 immune response	[94]
Live BCG	PLGA	Live BCG, DOX	Synergistic effect tumor ICD on BCG immunity. Enhanced DC activation and antigen presentation, activated APCs, and reprogrammed TAMs towards the M1	[95]

BCG: Bacillus Calmette–Guerin; NKG2D: natural-killer group 2, member D; CW: cell wall; BCG-CWS: Bacillus Calmette–Guerin cell wall skeleton; DC: dendritic cell; DOX: doxorubicin; ICD: immunogenic cell death; TAMs: tumor-associated macrophages; APCs: antigen-presenting cells.
